# Supplementary material for: Lumped circuit model for inductive antenna spin-wave transducers
Source: Sci Rep. 2022 Mar 8;12:3796. doi: 10.1038/s41598-022-07625-2 (PMC8904783; doi:10.1038/s41598-022-07625-2)
Supplement: Supplementary file 1 — Supplementary Information. [file 41598_2022_7625_MOESM1_ESM.pdf]

APPENDIX A: DYNAMIC SUSCEPTIBILITY

The magnetization dynamics can be described by the LLG equation [1, 2]

$$\frac{d\mathbf{M}}{dt} = -\gamma_0(\mathbf{M} \times \mathbf{H}_{\text{eff}}) + \frac{\alpha}{M_{\text{a}}}\left(\mathbf{M} \times \frac{d\mathbf{M}}{dt}\right) , \tag{A1}$$

with $\boldsymbol{M} = \boldsymbol{M}_0 + \boldsymbol{m}$, $\gamma_0 = |\gamma|\mu_0$, $|\gamma|$ the absolute value of the gyromagnetic ratio, $\boldsymbol{H}_{\text{eff}}$ the effective magnetic field and α the magnetic damping constant. $\boldsymbol{M}_0$ and $\boldsymbol{m}$ are the static and dynamic components of the magnetization $\mathbf{M}$, respectively. In this work, the effective field consist of a static bias field $\boldsymbol{H}_0$, a dynamic antenna field $\boldsymbol{h}_{\text{a}}$, spin-wave dipolar field $\boldsymbol{h}_{\text{d}}$ and spin-wave exchange field $\boldsymbol{h}_{\text{ex}}$. For weak magnetization dynamics, *i.e.* $|\boldsymbol{m}| \ll |\boldsymbol{M}_0|$, the LLG equation can be linearized and becomes

$$\begin{aligned} i\omega\boldsymbol{m}(\boldsymbol{k},\omega) = &- \gamma_0\left(\boldsymbol{M}_0 \times [\boldsymbol{h}_{\text{d}}(\boldsymbol{k},\omega) + \boldsymbol{h}_{\text{ex}}(\boldsymbol{k},\omega)] + \boldsymbol{M}_0 \times \boldsymbol{h}_{\text{a}}(\boldsymbol{k},\omega) + \boldsymbol{m}(\boldsymbol{k},\omega) \times \boldsymbol{H}_0\right) \\ &+ \frac{i\omega\alpha}{M_0}\left(\boldsymbol{M}_0 \times \boldsymbol{m}(\boldsymbol{k},\omega)\right) , \end{aligned} \tag{A2}$$

where complex notation was used and $\boldsymbol{k}$ and ω denote the spin-wave wavevector and angular frequency, respectively. The dynamic dipolar magnetic field is given by [3]

$$\boldsymbol{h}_{\text{d}}(\boldsymbol{r}) = \int\limits_V \hat{\Gamma}(\boldsymbol{r},\boldsymbol{r}')\boldsymbol{m}(\boldsymbol{r},\boldsymbol{r}')\, d\boldsymbol{r}' , \tag{A3}$$

with V the volume of the magnetic material and $\hat{\Gamma}(\boldsymbol{r},\boldsymbol{r}')$ the magnetostatic Green's function given by

$$\hat{\Gamma}(\boldsymbol{r},\boldsymbol{r}') = -\nabla_{\mathbf{r}}\nabla_{\mathbf{r}'}\frac{1}{|\mathbf{r}-\mathbf{r}'|} . \tag{A4}$$

For a plane wave in a thin film, the averaged dipolar magnetic field inside the film becomes [3–5]

$$\mathbf{h}_{\text{d}}(\mathbf{k},\omega) = -\begin{bmatrix} P\sin^2(\theta) & P\cos(\theta)\sin(\theta) & 0 \\ P\cos(\theta)\sin(\theta) & P\cos^2(\theta) & 0 \\ 0 & 0 & 1-P \end{bmatrix}\mathbf{m}(\mathbf{k},\omega) , \tag{A5}$$

with θ the (in-plane) angle between the static magnetization and the wavevector and

$$P = 1 - \frac{1-e^{-kt}}{kt} . \tag{A6}$$

The dynamic exchange field is given by

$$\mathbf{h}_{\text{ex}}(\mathbf{k},\omega) = \lambda_{\text{ex}}\nabla^2\boldsymbol{m}(\boldsymbol{r}) . \tag{A7}$$

For a plane wave, this becomes

$$\mathbf{h}_{\text{ex}}(\mathbf{k},\omega) = -\lambda_{\text{ex}} k^2 \mathbf{m}(\mathbf{k},\omega)\,, \tag{A8}$$

with

$$\lambda_{\text{ex}} = \sqrt{\frac{2A_{\text{ex}}}{\mu_0 M_{\text{a}}^2}} \tag{A9}$$

and A_{ex} the exchange stiffness constant. To simplify the expressions, the following tensor is introduced

$$\hat{F} = \begin{bmatrix} P\sin^2(\theta) + \lambda_{\text{ex}}k^2 & P\cos(\theta)\sin(\theta) & 0 \\ P\cos(\theta)\sin(\theta) & P\cos^2(\theta) + \lambda_{\text{ex}}k^2 & 0 \\ 0 & 0 & 1 - P + \lambda_{\text{ex}}k^2 \end{bmatrix}. \tag{A10}$$

The general form of the linearized LLG equation then becomes

$$\boldsymbol{m} = \hat{\chi}_\omega \boldsymbol{h}_a \tag{A11}$$

with

$$\hat{\chi}_\omega = \tag{A12}$$

$$-\omega_M \begin{bmatrix} i\omega + \omega_M F_{xy}\zeta_z & [\omega_0 + \omega_M F_{yy} + i\omega\alpha]\,\zeta_z & -[\omega_0 + \omega_M F_{zz} + i\omega\alpha]\,\zeta_y \\ [\omega_0 + \omega_M F_{xx} + i\omega\alpha]\,\zeta_z & i\omega + \omega_M F_{xy}\zeta_z & [\omega_0 + \omega_M F_{zz} + i\omega\alpha]\,\zeta_x \\ [\omega_0 + \omega_M F_{xx} + i\omega\alpha]\,\zeta_y - \omega_M F_{xy}\zeta_x & -[\omega_0 + \omega_M F_{yy} + i\omega\alpha]\,\zeta_x - \omega_M F_{xy}\zeta_y & i\omega \end{bmatrix}^{-1},$$

$\omega_M = \gamma_0 M_0$, $\omega_0 = \gamma_0 H_0$, and

$$\zeta = \begin{bmatrix} \cos(\psi)\cos(\theta) \\ \cos(\psi)\cos(\theta) \\ \sin(\psi) \end{bmatrix}. \tag{A13}$$

Here, ψ is the angle between the static magnetization $\boldsymbol{M}_0$ and the film. When the static magnetization direction coincides with one of the coordinate axes, the expression can be simplified and written as

$$\begin{bmatrix} \omega_1 + i\omega\alpha & -i\omega \\ i\omega & \omega_2 + i\omega\alpha \end{bmatrix} \begin{bmatrix} m_k \\ m_l \end{bmatrix} = \omega_{\text{M}} \begin{bmatrix} h_k \\ h_l \end{bmatrix}, \tag{A14}$$

which becomes

$$\begin{bmatrix} m_k \\ m_l \end{bmatrix} = \hat{\chi}_\omega \begin{bmatrix} h_k \\ h_l \end{bmatrix} \tag{A15}$$

$$= \frac{\omega_M}{(\omega_1\omega_2 - \omega^2) + i\alpha\omega\,(\omega_1+\omega_2)} \begin{bmatrix} \omega_2 + i\alpha\omega & i\omega \\ -i\omega & \omega_1 + i\alpha\omega \end{bmatrix} \begin{bmatrix} h_k \\ h_l \end{bmatrix} \tag{A16}$$

$$= \frac{\hat{\Omega}^2}{\omega_\mathrm{r}^2 - \omega^2 + i\omega\Gamma} \begin{bmatrix} h_k \\ h_l \end{bmatrix}, \tag{A17}$$

with $\omega_\mathrm{r} = \sqrt{\omega_1\omega_2}$ the spin-wave resonance frequency or dispersion relation and $\Gamma = \alpha\,(\omega_1+\omega_2)$ the spin-wave damping rate. The factors ω_1 and ω_2 are configuration dependent and are given for the three specific cases below. The susceptibility can be separated into a real and imaginary part as given below

$$\hat{\chi}_{kk} = \frac{\omega_M(\omega_2(\omega_r^2-\omega^2)+\alpha^2\omega^2(\omega_1+\omega_2))}{(\omega_\mathrm{r}^2-\omega^2)^2+\omega^2\alpha^2(\omega_1+\omega_2)^2} - i\frac{\alpha\omega\omega_M(\omega^2+\omega_2^2)}{(\omega_\mathrm{r}^2-\omega^2)^2+\omega^2\alpha^2(\omega_1+\omega_2)^2} \tag{A18}$$

$$\hat{\chi}_{ll} = \frac{\omega_M(\omega_1(\omega_r^2-\omega^2)+\alpha^2\omega^2(\omega_1+\omega_2))}{(\omega_\mathrm{r}^2-\omega^2)^2+\omega^2\alpha^2(\omega_1+\omega_2)^2} - i\frac{\alpha\omega\omega_M(\omega^2+\omega_1^2)}{(\omega_\mathrm{r}^2-\omega^2)^2+\omega^2\alpha^2(\omega_1+\omega_2)^2}\,. \tag{A19}$$

The group velocity is defined as

$$v_g = \frac{\partial\omega_r}{\partial k} = \frac{\partial\sqrt{\omega_1\omega_2}}{\partial k} = \frac{1}{2\omega_1\omega_2}\left[\omega_2\frac{\partial\omega_1}{\partial k} + \omega_1\frac{\partial\omega_2}{\partial k}\right]. \tag{A20}$$

1. Specific cases

For $\theta = 0$ and $\psi = 0$ (backward-volume configuration), $k = y$, $l = z$ and

$$\begin{cases} \omega_1 = \omega_0 + \omega_M\left(\lambda_\mathrm{ex}k^2\right) \\ \omega_2 = \omega_0 + \omega_M\left(\lambda_\mathrm{ex}k^2 + 1 - P\right) \end{cases}.$$

For $\theta = \pi/2$ and $\psi = 0$ (Damon–Eshbach configuration), $k = z$, $l = x$ and

$$\begin{cases} \omega_1 = \omega_0 + \omega_M\left(\lambda_\mathrm{ex}k^2 + 1 - P\right) \\ \omega_2 = \omega_0 + \omega_M\left(\lambda_\mathrm{ex}k^2 + P\right) \end{cases}.$$

For $\phi = \pi/2$ (forward-volume configuration), $k = x$, $l = y$ and

$$\begin{cases} \omega_1 = \omega_0 + \omega_M\left(\lambda_\mathrm{ex}k^2 + P\right) \\ \omega_2 = \omega_0 + \omega_M\left(\lambda_\mathrm{ex}k^2\right) \end{cases}.$$

II. REFERENCES

[1] L. Landau, E. Lifshitz, and L.P. Pitaevski, *On the theory of the dispersion of magnetic permeability in ferromagnetic bodies*, in *Perspectives in Theoretical Physics*, pp. 51 (Pergamon, Oxford, 1992).

[2] T.L. Gilbert, IEEE Trans. Magn. **40**, 3443 (2004).

[3] A.G. Gurevich and G.A. Melkov, *Magnetization Oscillations and Waves* (CRC Press, Boca Raton, 1996).

[4] R.W. Damon and J.R. Eshbach, J. Appl. Phys. **31**, S104 (1960).

[5] K.J. Harte, J. Appl. Phys. **39**, 1503 (1968).
